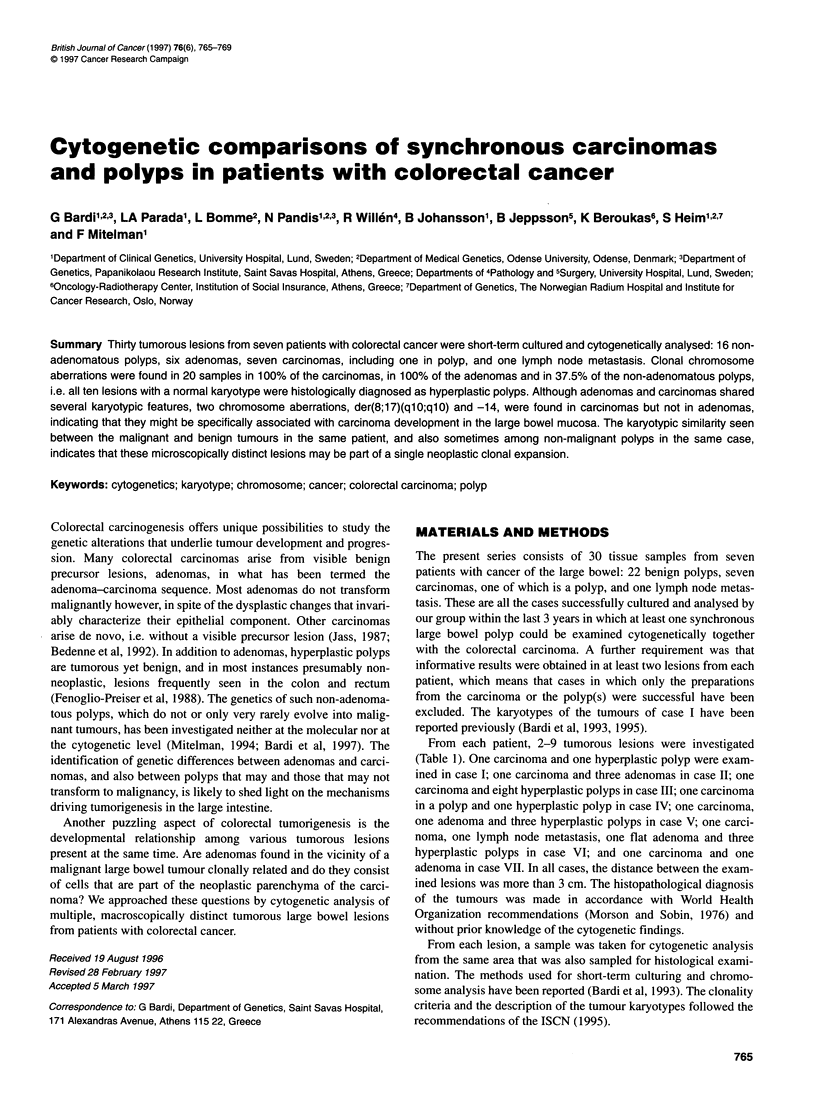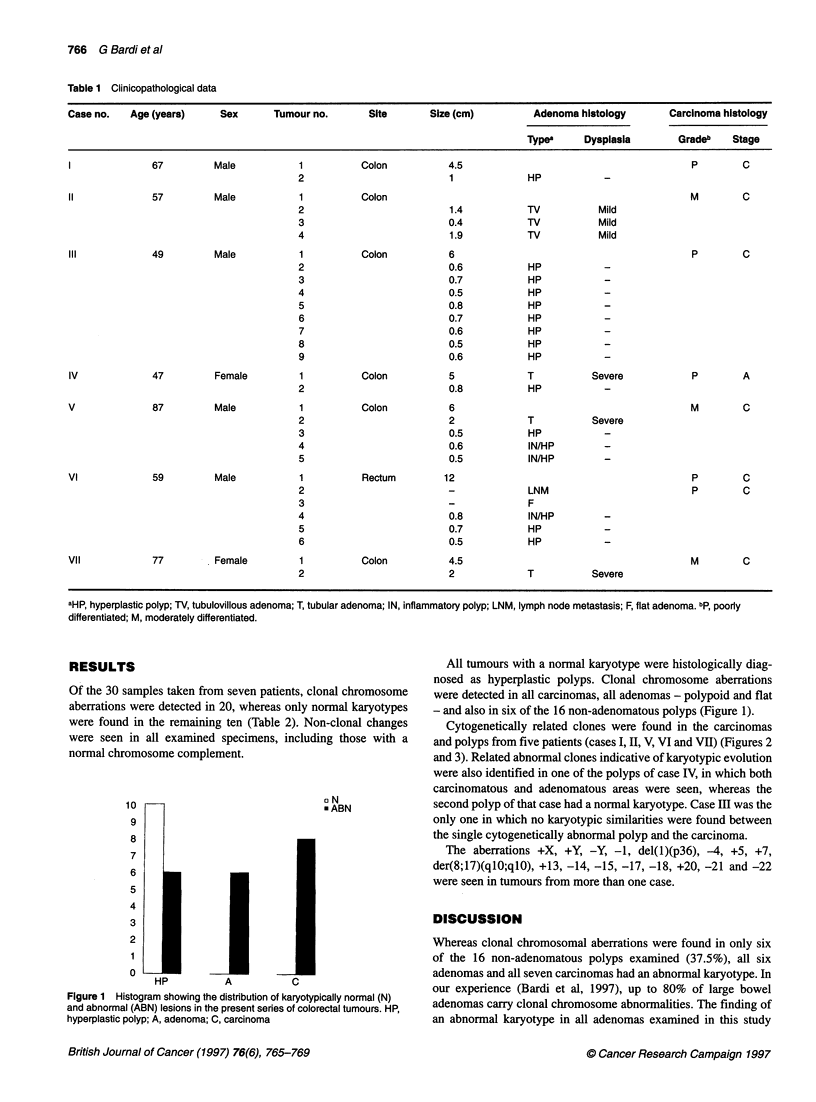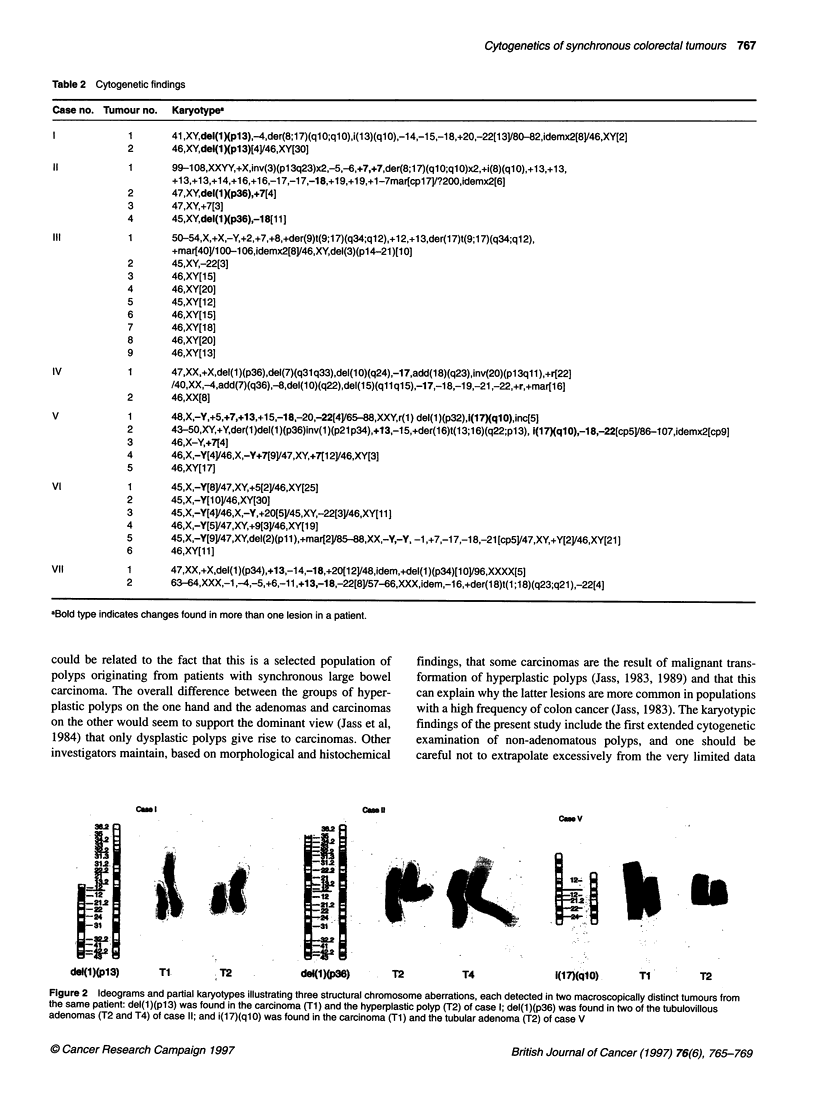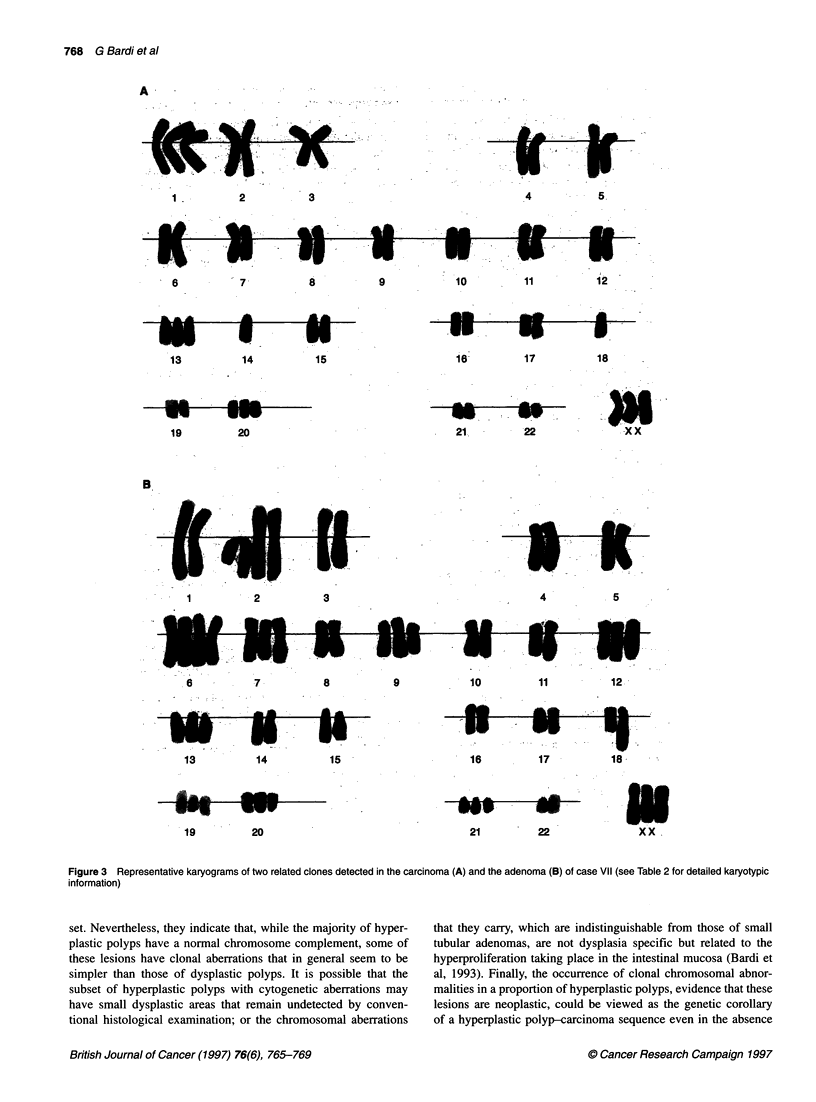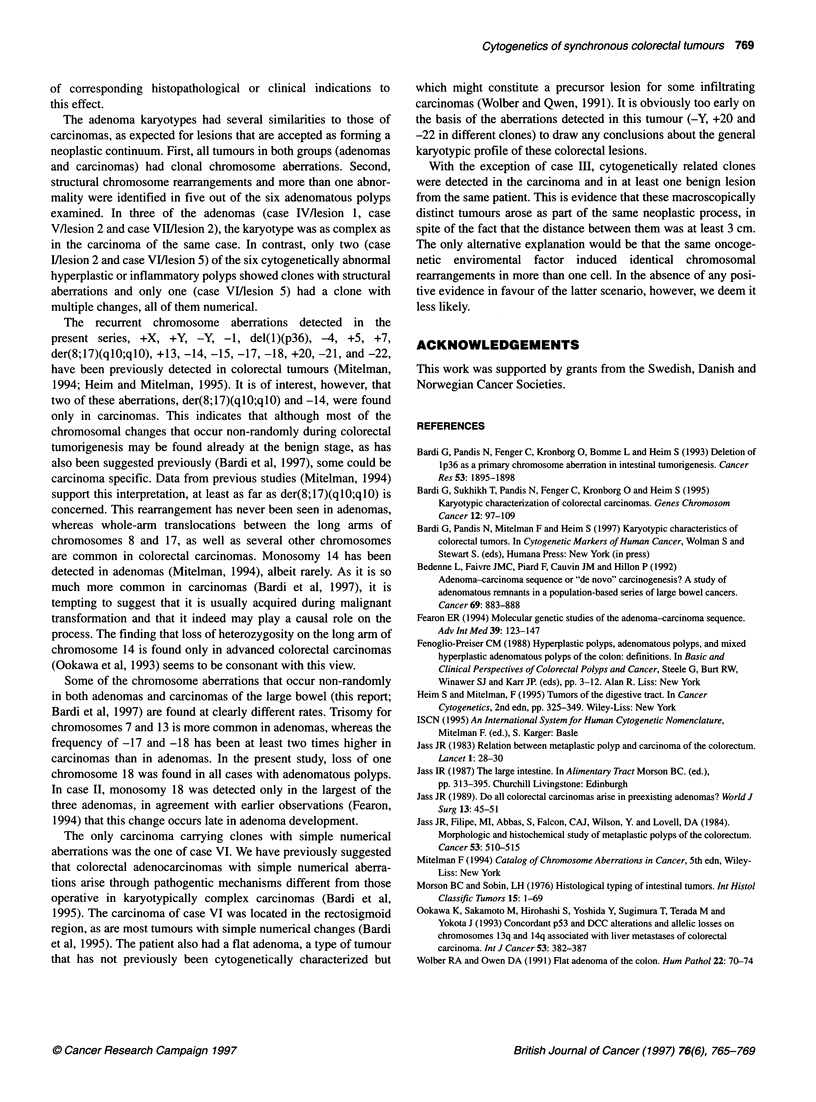# Cytogenetic comparisons of synchronous carcinomas and polyps in patients with colorectal cancer.

**DOI:** 10.1038/bjc.1997.459

**Published:** 1997

**Authors:** G. Bardi, L. A. Parada, L. Bomme, N. Pandis, R. WillÃ©n, B. Johansson, B. Jeppsson, K. Beroukas, S. Heim, F. Mitelman

**Affiliations:** Department of Clinical Genetics, University Hospital, Lund, Sweden.

## Abstract

**Images:**


British Journal of Cancer (1997) 76(6), 765–769
© 1997 Cancer Research Campaign

### Cytogenetic comparisons of synchronous carcinomas and polyps in patients with colorectal cancer

G Bardi[1,2,3], LA Parada[1], L Bomme[2], N Pandis[1,2,3], R Willén[4], B Johansson[1], B Jeppsson[5], K Beroukas[6], S Heim[1,2,7] and F Mitelman[1]

[1]Department of Clinical Genetics, University Hospital, Lund, Sweden; [2]Department of Medical Genetics, Odense University, Odense, Denmark; [3]Department of Genetics, Papanikolaou Research Institute, Saint Savas Hospital, Athens, Greece; Departments of [4]Pathology and [5]Surgery, University Hospital, Lund, Sweden; [6]Oncology-Radiotherapy Center, Institution of Social Insurance, Athens, Greece; [7]Department of Genetics, The Norwegian Radium Hospital and Institute for Cancer Research, Oslo, Norway

**Summary** Thirty tumorous lesions from seven patients with colorectal cancer were short-term cultured and cytogenetically analysed: 16 non-adenomatous polyps, six adenomas, seven carcinomas, including one in polyp, and one lymph node metastasis. Clonal chromosome aberrations were found in 20 samples in 100% of the carcinomas, in 100% of the adenomas and in 37.5% of the non-adenomatous polyps, i.e. all ten lesions with a normal karyotype were histologically diagnosed as hyperplastic polyps. Although adenomas and carcinomas shared several karyotypic features, two chromosome aberrations, der(8;17)(q10;q10) and −14, were found in carcinomas but not in adenomas, indicating that they might be specifically associated with carcinoma development in the large bowel mucosa. The karyotypic similarity seen between the malignant and benign tumours in the same patient, and also sometimes among non-malignant polyps in the same case, indicates that these microscopically distinct lesions may be part of a single neoplastic clonal expansion.

**Keywords:** cytogenetics; karyotype; chromosome; cancer; colorectal carcinoma; polyp

Colorectal carcinogenesis offers unique possibilities to study the genetic alterations that underlie tumour development and progression. Many colorectal carcinomas arise from visible benign precursor lesions, adenomas, in what has been termed the adenoma–carcinoma sequence. Most adenomas do not transform malignantly however, in spite of the dysplastic changes that invariably characterize their epithelial component. Other carcinomas arise de novo, i.e. without a visible precursor lesion (Jass, 1987; Bedenne et al, 1992). In addition to adenomas, hyperplastic polyps are tumorous yet benign, and in most instances presumably non-neoplastic, lesions frequently seen in the colon and rectum (Fenoglio-Preiser et al, 1988). The genetics of such non-adenomatous polyps, which do not or only very rarely evolve into malignant tumours, has been investigated neither at the molecular nor at the cytogenetic level (Mitelman, 1994; Bardi et al, 1997). The identification of genetic differences between adenomas and carcinomas, and also between polyps that may and those that may not transform to malignancy, is likely to shed light on the mechanisms driving tumorigenesis in the large intestine.

Another puzzling aspect of colorectal tumorigenesis is the developmental relationship among various tumorous lesions present at the same time. Are adenomas found in the vicinity of a malignant large bowel tumour clonally related and do they consist of cells that are part of the neoplastic parenchyma of the carcinoma? We approached these questions by cytogenetic analysis of multiple, macroscopically distinct tumorous large bowel lesions from patients with colorectal cancer.

*Received 19 August 1996*
*Revised 28 February 1997*
*Accepted 5 March 1997*

*Correspondence to:* G Bardi, Department of Genetics, Saint Savas Hospital, 171 Alexandras Avenue, Athens 115 22, Greece

### MATERIALS AND METHODS

The present series consists of 30 tissue samples from seven patients with cancer of the large bowel: 22 benign polyps, seven carcinomas, one of which is a polyp, and one lymph node metastasis. These are all the cases successfully cultured and analysed by our group within the last 3 years in which at least one synchronous large bowel polyp could be examined cytogenetically together with the colorectal carcinoma. A further requirement was that informative results were obtained in at least two lesions from each patient, which means that cases in which only the preparations from the carcinoma or the polyp(s) were successful have been excluded. The karyotypes of the tumours of case I have been reported previously (Bardi et al, 1993, 1995).

From each patient, 2–9 tumorous lesions were investigated (Table 1). One carcinoma and one hyperplastic polyp were examined in case I; one carcinoma and three adenomas in case II; one carcinoma and eight hyperplastic polyps in case III; one carcinoma in a polyp and one hyperplastic polyp in case IV; one carcinoma, one adenoma and three hyperplastic polyps in case V; one carcinoma, one lymph node metastasis, one flat adenoma and three hyperplastic polyps in case VI; and one carcinoma and one adenoma in case VII. In all cases, the distance between the examined lesions was more than 3 cm. The histopathological diagnosis of the tumours was made in accordance with World Health Organization recommendations (Morson and Sobin, 1976) and without prior knowledge of the cytogenetic findings.

From each lesion, a sample was taken for cytogenetic analysis from the same area that was also sampled for histological examination. The methods used for short-term culturing and chromosome analysis have been reported (Bardi et al, 1993). The clonality criteria and the description of the tumour karyotypes followed the recommendations of the ISCN (1995).

**Table 1** Clinicopathological data

| Case no. | Age (years) | Sex | Tumour no. | Site | Size (cm) | Adenoma histology | | Carcinoma histology | |
|---|---|---|---|---|---|---|---|---|---|
| | | | | | | Type[a] | Dysplasia | Grade[b] | Stage |
| I | 67 | Male | 1 | Colon | 4.5 | | | P | C |
| | | | 2 | | 1 | HP | – | | |
| II | 57 | Male | 1 | Colon | | | | M | C |
| | | | 2 | | 1.4 | TV | Mild | | |
| | | | 3 | | 0.4 | TV | Mild | | |
| | | | 4 | | 1.9 | TV | Mild | | |
| III | 49 | Male | 1 | Colon | 6 | | | P | C |
| | | | 2 | | 0.6 | HP | – | | |
| | | | 3 | | 0.7 | HP | – | | |
| | | | 4 | | 0.5 | HP | – | | |
| | | | 5 | | 0.8 | HP | – | | |
| | | | 6 | | 0.7 | HP | – | | |
| | | | 7 | | 0.6 | HP | – | | |
| | | | 8 | | 0.5 | HP | – | | |
| | | | 9 | | 0.6 | HP | – | | |
| IV | 47 | Female | 1 | Colon | 5 | T | Severe | P | A |
| | | | 2 | | 0.8 | HP | – | | |
| V | 87 | Male | 1 | Colon | 6 | | | M | C |
| | | | 2 | | 2 | T | Severe | | |
| | | | 3 | | 0.5 | HP | – | | |
| | | | 4 | | 0.6 | IN/HP | – | | |
| | | | 5 | | 0.5 | IN/HP | – | | |
| VI | 59 | Male | 1 | Rectum | 12 | | | P | C |
| | | | 2 | | – | LNM | | P | C |
| | | | 3 | | – | F | | | |
| | | | 4 | | 0.8 | IN/HP | – | | |
| | | | 5 | | 0.7 | HP | – | | |
| | | | 6 | | 0.5 | HP | – | | |
| VII | 77 | Female | 1 | Colon | 4.5 | | | M | C |
| | | | 2 | | 2 | T | Severe | | |

[a]HP, hyperplastic polyp; TV, tubulovillous adenoma; T, tubular adenoma; IN, inflammatory polyp; LNM, lymph node metastasis; F, flat adenoma. [b]P, poorly differentiated; M, moderately differentiated.

### RESULTS

Of the 30 samples taken from seven patients, clonal chromosome aberrations were detected in 20, whereas only normal karyotypes were found in the remaining ten (Table 2). Non-clonal changes were seen in all examined specimens, including those with a normal chromosome complement.

**Figure 1** Histogram showing the distribution of karyotypically normal (N) and abnormal (ABN) lesions in the present series of colorectal tumours. HP, hyperplastic polyp; A, adenoma; C, carcinoma

All tumours with a normal karyotype were histologically diagnosed as hyperplastic polyps. Clonal chromosome aberrations were detected in all carcinomas, all adenomas – polypoid and flat – and also in six of the 16 non-adenomatous polyps (Figure 1).

Cytogenetically related clones were found in the carcinomas and polyps from five patients (cases I, II, V, VI and VII) (Figures 2 and 3). Related abnormal clones indicative of karyotypic evolution were also identified in one of the polyps of case IV, in which both carcinomatous and adenomatous areas were seen, whereas the second polyp of that case had a normal karyotype. Case III was the only one in which no karyotypic similarities were found between the single cytogenetically abnormal polyp and the carcinoma.

The aberrations +X, +Y, –Y, –1, del(1)(p36), –4, +5, +7, der(8;17)(q10;q10), +13, –14, –15, –17, –18, +20, –21 and –22 were seen in tumours from more than one case.

### DISCUSSION

Whereas clonal chromosomal aberrations were found in only six of the 16 non-adenomatous polyps examined (37.5%), all six adenomas and all seven carcinomas had an abnormal karyotype. In our experience (Bardi et al, 1997), up to 80% of large bowel adenomas carry clonal chromosome abnormalities. The finding of an abnormal karyotype in all adenomas examined in this study

© Cancer Research Campaign 1997

**Table 2** Cytogenetic findings

| Case no. | Tumour no. | Karyotype[a] |
|---|---|---|
| I | 1 | 41,XY,**del(1)(p13)**,–4,der(8;17)(q10;q10),i(13)(q10),–14,–15,–18,+20,–22[13]/80–82,idemx2[8]/46,XY[2] |
| | 2 | 46,XY,**del(1)(p13)**[4]/46,XY[30] |
| II | 1 | 99–108,XXYY,+X,inv(3)(p13q23)x2,–5,–6,**+7,+7**,der(8;17)(q10;q10)x2,+i(8)(q10),+13,+13, +13,+13,+14,+16,+16,–17,–17,**–18**,+19,+19,+1–7mar[cp17]/?200,idemx2[6] |
| | 2 | 47,XY,**del(1)(p36),+7**[4] |
| | 3 | 47,XY,+7[3] |
| | 4 | 45,XY,**del(1)(p36),–18**[11] |
| III | 1 | 50–54,X,+X,–Y,+2,+7,+8,+der(9)t(9;17)(q34;q12),+12,+13,der(17)t(9;17)(q34;q12), +mar[40]/100–106,idemx2[8]/46,XY,del(3)(p14–21)[10] |
| | 2 | 45,XY,–22[3] |
| | 3 | 46,XY[15] |
| | 4 | 46,XY[20] |
| | 5 | 45,XY[12] |
| | 6 | 46,XY[15] |
| | 7 | 46,XY[18] |
| | 8 | 46,XY[20] |
| | 9 | 46,XY[13] |
| IV | 1 | 47,XX,+X,del(1)(p36),del(7)(q31q33),del(10)(q24),**–17**,add(18)(q23),inv(20)(p13q11),+**r**[22] /40,XX,–4,add(7)(q36),–8,del(10)(q22),del(15)(q11q15),**–17**,–18,–19,–21,–22,+**r**,+mar[16] |
| | 2 | 46,XX[8] |
| V | 1 | 48,X,**–Y**,+5,**+7,+13**,+15,**–18**,–20,**–22**[4]/65–88,XXY,r(1) del(1)(p32),**i(17)(q10)**,inc[5] |
| | 2 | 43–50,XY,+Y,der(1)del(1)(p36)inv(1)(p21p34),**+13**,–15,+der(16)t(13;16)(q22;p13), **i(17)(q10),–18,–22**[cp5]/86–107,idemx2[cp9] |
| | 3 | 46,X–Y,**+7**[4] |
| | 4 | 46,X,**–Y**[4]/46,X,**–Y+7**[9]/47,XY,**+7**[12]/46,XY[3] |
| | 5 | 46,XY[17] |
| VI | 1 | 45,X,**–Y**[8]/47,XY,+5[2]/46,XY[25] |
| | 2 | 45,X,**–Y**[10]/46,XY[30] |
| | 3 | 45,X,**–Y**[4]/46,X,**–Y**,+20[5]/45,XY,–22[3]/46,XY[11] |
| | 4 | 46,X,**–Y**[5]/47,XY,+9[3]/46,XY[19] |
| | 5 | 45,X,**–Y**[9]/47,XY,del(2)(p11),+mar[2]/85–88,XX,**–Y,–Y**, –1,+7,–17,–18,–21[cp5]/47,XY,+Y[2]/46,XY[21] |
| | 6 | 46,XY[11] |
| VII | 1 | 47,XX,+X,del(1)(p34),**+13**,–14,**–18**,+20[12]/48,idem,+del(1)(p34)[10]/96,XXXX[5] |
| | 2 | 63–64,XXX,–1,–4,–5,+6,–11,**+13,–18**,–22[8]/57–66,XXX,idem,–16,+der(18)t(1;18)(q23;q21),–22[4] |

[a]Bold type indicates changes found in more than one lesion in a patient.

could be related to the fact that this is a selected population of polyps originating from patients with synchronous large bowel carcinoma. The overall difference between the groups of hyperplastic polyps on the one hand and the adenomas and carcinomas on the other would seem to support the dominant view (Jass et al, 1984) that only dysplastic polyps give rise to carcinomas. Other investigators maintain, based on morphological and histochemical findings, that some carcinomas are the result of malignant transformation of hyperplastic polyps (Jass, 1983, 1989) and that this can explain why the latter lesions are more common in populations with a high frequency of colon cancer (Jass, 1983). The karyotypic findings of the present study include the first extended cytogenetic examination of non-adenomatous polyps, and one should be careful not to extrapolate excessively from the very limited data

**Figure 2** Ideograms and partial karyotypes illustrating three structural chromosome aberrations, each detected in two macroscopically distinct tumours from the same patient: del(1)(p13) was found in the carcinoma (T1) and the hyperplastic polyp (T2) of case I; del(1)(p36) was found in two of the tubulovillous adenomas (T2 and T4) of case II; and i(17)(q10) was found in the carcinoma (T1) and the tubular adenoma (T2) of case V

© *Cancer Research Campaign 1997* 

**Figure 3** Representative karyograms of two related clones detected in the carcinoma (**A**) and the adenoma (**B**) of case VII (see Table 2 for detailed karyotypic information)

set. Nevertheless, they indicate that, while the majority of hyperplastic polyps have a normal chromosome complement, some of these lesions have clonal aberrations that in general seem to be simpler than those of dysplastic polyps. It is possible that the subset of hyperplastic polyps with cytogenetic aberrations may have small dysplastic areas that remain undetected by conventional histological examination; or the chromosomal aberrations that they carry, which are indistinguishable from those of small tubular adenomas, are not dysplasia specific but related to the hyperproliferation taking place in the intestinal mucosa (Bardi et al, 1993). Finally, the occurrence of clonal chromosomal abnormalities in a proportion of hyperplastic polyps, evidence that these lesions are neoplastic, could be viewed as the genetic corollary of a hyperplastic polyp–carcinoma sequence even in the absence

© Cancer Research Campaign 1997

of corresponding histopathological or clinical indications to this effect.

The adenoma karyotypes had several similarities to those of carcinomas, as expected for lesions that are accepted as forming a neoplastic continuum. First, all tumours in both groups (adenomas and carcinomas) had clonal chromosome aberrations. Second, structural chromosome rearrangements and more than one abnormality were identified in five out of the six adenomatous polyps examined. In three of the adenomas (case IV/lesion 1, case V/lesion 2 and case VII/lesion 2), the karyotype was as complex as in the carcinoma of the same case. In contrast, only two (case I/lesion 2 and case VI/lesion 5) of the six cytogenetically abnormal hyperplastic or inflammatory polyps showed clones with structural aberrations and only one (case VI/lesion 5) had a clone with multiple changes, all of them numerical.

The recurrent chromosome aberrations detected in the present series, +X, +Y, –Y, –1, del(1)(p36), –4, +5, +7, der(8;17)(q10;q10), +13, –14, –15, –17, –18, +20, –21, and –22, have been previously detected in colorectal tumours (Mitelman, 1994; Heim and Mitelman, 1995). It is of interest, however, that two of these aberrations, der(8;17)(q10;q10) and –14, were found only in carcinomas. This indicates that although most of the chromosomal changes that occur non-randomly during colorectal tumorigenesis may be found already at the benign stage, as has also been suggested previously (Bardi et al, 1997), some could be carcinoma specific. Data from previous studies (Mitelman, 1994) support this interpretation, at least as far as der(8;17)(q10;q10) is concerned. This rearrangement has never been seen in adenomas, whereas whole-arm translocations between the long arms of chromosomes 8 and 17, as well as several other chromosomes are common in colorectal carcinomas. Monosomy 14 has been detected in adenomas (Mitelman, 1994), albeit rarely. As it is so much more common in carcinomas (Bardi et al, 1997), it is tempting to suggest that it is usually acquired during malignant transformation and that it indeed may play a causal role on the process. The finding that loss of heterozygosity on the long arm of chromosome 14 is found only in advanced colorectal carcinomas (Ookawa et al, 1993) seems to be consonant with this view.

Some of the chromosome aberrations that occur non-randomly in both adenomas and carcinomas of the large bowel (this report; Bardi et al, 1997) are found at clearly different rates. Trisomy for chromosomes 7 and 13 is more common in adenomas, whereas the frequency of –17 and –18 has been at least two times higher in carcinomas than in adenomas. In the present study, loss of one chromosome 18 was found in all cases with adenomatous polyps. In case II, monosomy 18 was detected only in the largest of the three adenomas, in agreement with earlier observations (Fearon, 1994) that this change occurs late in adenoma development.

The only carcinoma carrying clones with simple numerical aberrations was the one of case VI. We have previously suggested that colorectal adenocarcinomas with simple numerical aberrations arise through pathogentic mechanisms different from those operative in karyotypically complex carcinomas (Bardi et al, 1995). The carcinoma of case VI was located in the rectosigmoid region, as are most tumours with simple numerical changes (Bardi et al, 1995). The patient also had a flat adenoma, a type of tumour that has not previously been cytogenetically characterized but which might constitute a precursor lesion for some infiltrating carcinomas (Wolber and Qwen, 1991). It is obviously too early on the basis of the aberrations detected in this tumour (–Y, +20 and –22 in different clones) to draw any conclusions about the general karyotypic profile of these colorectal lesions.

With the exception of case III, cytogenetically related clones were detected in the carcinoma and in at least one benign lesion from the same patient. This is evidence that these macroscopically distinct tumours arose as part of the same neoplastic process, in spite of the fact that the distance between them was at least 3 cm. The only alternative explanation would be that the same oncogenetic enviromental factor induced identical chromosomal rearrangements in more than one cell. In the absence of any positive evidence in favour of the latter scenario, however, we deem it less likely.

### ACKNOWLEDGEMENTS

This work was supported by grants from the Swedish, Danish and Norwegian Cancer Societies.

### REFERENCES

Bardi G, Pandis N, Fenger C, Kronborg O, Bomme L and Heim S (1993) Deletion of 1p36 as a primary chromosome aberration in intestinal tumorigenesis. *Cancer Res* **53**: 1895–1898

Bardi G, Sukhikh T, Pandis N, Fenger C, Kronborg O and Heim S (1995) Karyotypic characterization of colorectal carcinomas. *Genes Chromosom Cancer* **12**: 97–109

Bardi G, Pandis N, Mitelman F and Heim S (1997) Karyotypic characteristics of colorectal tumors. In *Cytogenetic Markers of Human Cancer*, Wolman S and Stewart S. (eds), Humana Press: New York (in press)

Bedenne L, Faivre JMC, Piard F, Cauvin JM and Hillon P (1992) Adenoma–carcinoma sequence or "de novo" carcinogenesis? A study of adenomatous remnants in a population-based series of large bowel cancers. *Cancer* **69**: 883–888

Fearon ER (1994) Molecular genetic studies of the adenoma–carcinoma sequence. *Adv Int Med* **39**: 123–147

Fenoglio-Preiser CM (1988) Hyperplastic polyps, adenomatous polyps, and mixed hyperplastic adenomatous polyps of the colon: definitions. In *Basic and Clinical Perspectives of Colorectal Polyps and Cancer*, Steele G, Burt RW, Winawer SJ and Karr JP. (eds), pp. 3–12. Alan R. Liss: New York

Heim S and Mitelman, F (1995) Tumors of the digestive tract. In *Cancer Cytogenetics*, 2nd edn, pp. 325–349. Wiley-Liss: New York

ISCN (1995) *An International System for Human Cytogenetic Nomenclature*, Mitelman F. (ed.), S. Karger: Basle

Jass JR (1983) Relation between metaplastic polyp and carcinoma of the colorectum. *Lancet* **1**: 28–30

Jass IR (1987) The large intestine. In *Alimentary Tract* Morson BC. (ed.), pp. 313–395. Churchill Livingstone: Edinburgh

Jass JR (1989). Do all colorectal carcinomas arise in preexisting adenomas? *World J Surg* **13**: 45–51

Jass JR, Filipe, MI, Abbas, S, Falcon, CAJ, Wilson, Y. and Lovell, DA (1984). Morphologic and histochemical study of metaplastic polyps of the colorectum. *Cancer* **53**: 510–515

Mitelman F (1994) *Catalog of Chromosome Aberrations in Cancer*, 5th edn, Wiley-Liss: New York

Morson BC and Sobin, LH (1976) Histological typing of intestinal tumors. *Int Histol Classific Tumors* **15**: 1–69

Ookawa K, Sakamoto M, Hirohashi S, Yoshida Y, Sugimura T, Terada M and Yokota J (1993) Concordant p53 and DCC alterations and allelic losses on chromosomes 13q and 14q associated with liver metastases of colorectal carcinoma. *Int J Cancer* **53**: 382–387

Wolber RA and Owen DA (1991) Flat adenoma of the colon. *Hum Pathol* **22**: 70–74

© Cancer Research Campaign 1997